# Relation between platelet coagulant and vascular function, sex-specific analysis in adult survivors of childhood cancer compared to a population-based sample

**DOI:** 10.1038/s41598-019-56626-1

**Published:** 2019-12-27

**Authors:** Marina Panova-Noeva, Bianca Wagner, Markus Nagler, Natalie Arnold, Jürgen H. Prochaska, Susan Eckerle, Henri M. Spronk, Hiltrud Merzenich, Arthur Wingerter, Astrid Schneider, Sven Danckwardt, Hugo ten Cate, Jörg Faber, Philipp S. Wild

**Affiliations:** 1 Center for Thrombosis and Hemostasis (CTH), University Medical Center of the Johannes Gutenberg-University Mainz, Mainz, Germany; 2 Preventive Cardiology and Preventive Medicine, Center for Cardiology, University Medical Center of the Johannes Gutenberg-University Mainz, Mainz, Germany; 3 Department of Pediatric Hematology/Oncology/Hemostaseology, Center for Pediatric and Adolescent Medicine, University Medical Center of the Johannes Gutenberg-University Mainz, Mainz, Germany; 4 Laboratory for Clinical Thrombosis and Hemostasis, Department of Internal Medicine, Cardiovascular Research Institute Maastricht (CARIM), Maastricht University Medical Center, Maastricht, The Netherlands; 5 Institute of Medical Biostatistics, Epidemiology and Informatics (IMBEI), University Medical Center of the Johannes Gutenberg-University Mainz, Mainz, Germany; 6 Institute for Clinical Chemistry and Laboratory Medicine, University Medical Center of the Johannes Gutenberg-University Mainz, Mainz, Germany; 7 DZHK (German Center for Cardiovascular Research), Partner Site RhineMain, Mainz, Germany

**Keywords:** Hypertension, Epidemiology

## Abstract

Female sex is a risk factor for long-term adverse outcome in cancer survivors, however very little is known for the underlying pathophysiological mechanisms rendering the increased risk. This study investigated sex-specifically the relation between thrombin generation (TG) with and without presence of platelets and vascular function in 200 adult survivors of a childhood cancer compared to 335 population-based control individuals. TG lag time, peak height and endogenous thrombin potential (ETP) measured in presence and absence of platelets were correlated to reflection index (RI) and stiffness index (SI). A sex-specific correlation analysis showed a negative relation in female survivors for platelet-dependent peak height and/or ETP and RI only. An age adjusted linear regression model confirmed the negative association between RI and platelet-dependent ETP (beta estimate: −6.85, 95% confidence interval: −12.19,−1.51) in females. Adjustment for cardiovascular risk factors resulted in loss of the association, whereby arterial hypertension and obesity showed the largest effects on the observed association. No other relevant associations were found in male and female cancer survivors and all population-based controls. This study demonstrates a link between platelet coagulant and vascular function of resistance vessels, found in female cancer survivors, potentially mediated by the presence of arterial hypertension and obesity.

## Introduction

Improvement of childhood cancer treatment over the last decades has resulted in steady increase in childhood cancer survivors^1^. Unfortunately, adult childhood cancer survivors manifest excess cardiovascular risk throughout survivorship with circulatory diseases overtaking from subsequent neoplasms the leading cause of excess mortality^2,3^. The underlying pathophysiological mechanism for an increased cardiovascular risk in this vulnerable population is not well elucidated yet. Ischemic heart disease followed by cardiomyopathies and heart failure have been reported as the leading causes of cardiac deaths^2^.

Enhanced coagulation increases the risk for cardiovascular diseases (CVD). Both inflammation and endothelial dysfunction can upregulate blood coagulation towards a hypercoagulable state^4,5^. Thrombin generation (TG) assays have emerged as important coagulation assays that can increase the understanding for the link between the coagulation system and CVD^6^. In subjects with acute coronary syndrome, the increased TG potential has been associated to recurrent cardiovascular events^7^. Our recent work highlights the relevance of platelet-dependent TG as potential tool to evaluate individual risk for CVD^8^. In addition to hypercoagulability, vascular dysfunction is another important factor implicated in CVD development and progression^9^. Vascular endothelium regulates vessel homeostasis by release of nitric oxide through inhibiting vascular cell proliferation, enhancing vasodilation and inhibiting platelet adhesion and aggregation. Treatment-related beneficial effects on vascular endothelial function has been associated with improvement of the disease process and reduced adverse outcomes^10^. Vascular toxicity, including both abnormal functional and structural properties of the vasculature, as a consequence of oncological treatment has been recognized in cancer survivors^11^.

Our recent study reported on premature occurrence of arterial hypertension and dyslipidemia in adult survivors of childhood cancer compared to age and sex matched control individuals from the population-based study^12^. In addition, in a representative subsample from the adult survivors of childhood cancer cohort, a platelet-dependent hypercoagulability for individuals with obesity and arterial hypertension was observed. The sex-specific analysis demonstrated that females presented with increased TG potential compared to male cancer survivors, both in presence and absence of platelets^13^. Whether platelet-dependent hypercoagulability is associated with endothelial dysfunction presently is unknown. This study aims to investigate the association between TG in presence and absence of platelets and vascular function, sex specifically, in cancer survivors from the Cardiac and Vascular late Sequelae in long-term Survivors of childhood cancer (CVSS)-study. The sex-specific relation between TG and vascular function was also investigated in control individuals from the population-based Gutenberg Health Study (GHS).

## Results

Demographic, clinical and laboratory characteristics of the investigated sample are presented in Table 1. Compared to the whole cohort, published elsewhere^12^, investigated study subsample had comparable age, sex and presence of conventional cardiovascular risk factors (CVRFs). Female cancer survivors presented with higher platelet and leukocyte counts, lower low density lipoprotein cholesterol/high density lipoprotein cholesterol (LDL/HDL) ratio and higher C-reactive protein (CRP) and fibrinogen levels compared to male cancer survivors. The sex-specific characteristics of the control subjects are presented in the Supplemental Table S1.

**Table 1:** Characteristics of the cancer survivor sample.

	Whole sample	Males	Females	p-value
Number (N)	200	115	85	—
Sex (females), % (N)	42.5 (85)	—	—	—
Age (years), mean (SD)	35.1 (5.2)	35.5 (5.4)	34.6 (5.1)	0.23
**Traditional Cardiovascular Risk Factors**				
Obesity, % (N)	20.5 (41)	15.7 (18)	27.1 (23)	0.053
Smoking, % (N)	21.2 (42)	21.9 (25)	20.2(17)	0.86
Diabetes mellitus, % (N)	1.0(2)	0 (0)	2.4(2)	0.18
Arterial Hypertension, % (N)	23.1 (46)	25.2 (29)	20.2 (17)	0.50
Dyslipidemia, % (N)	30.0(60)	37.4 (43)	20.0 (17)	0.0083
**Standard laboratory**				
Platelet count (x10^9^/l), mean (SD)	242 (56)	223 (45)	267 (60)	<0.0001
Mean platelet volume (fl), median (25%/75% Q)	7.30 (7.00/7.70)	7.30 (6.90/7.68)	7.30 (7.00/7.80)	0.31
Leukocyte count (x10^9^/l), mean (SD)	6.98 (1.95)	6.39 (1.58)	7.79 (2.10)	<0.0001
Cholesterol (mg/dl), mean (SD)	207 (39)	209 (37)	205 (43)	0.59
Triglycerides (mg/dl), median (25%/75% Q)	104.0 (74.0/133.2)	108.0 (84.3/140.7)	89.0 (65.0/119.7)	0.015
LDL/HDL ratio, mean (SD)	2.62 (1.02)	3.01 (1.01)	2.10 (0.77)	<0.0001
HbA1c (%), mean/(SD)	5.26 (0.33)	5.28 (0.31)	5.24 (0.35)	0.42
C-reactive protein (mg/l), median (25%/75% Q)	1.45 (0.67/3.88)	1.20 (0.56/2.50)	2.40 (0.82/5.40)	0.0031
Fibrinogen (mg/dl), mean (SD)	291 (76)	268 (61)	322 (83)	<0.0001
**Vascular function**				
Reflection index (%), median (25%/75% Q)	64.0 (50.0/77.0)	71.0 (55.0/81.0)	52.0 (43.0/64.2)	<0.0001
Stiffness index (m/s), median (25%/75% Q)	6.31 (5.57/7.58)	6.65 (5.95/8.26)	5.76 (5.01/7.02)	<0.0001
**Thrombin generation**				
PRP_Lag time (min), median (25%/75% Q)	6.17 (5.46/7.33)	6.78 (5.83/8.00)	5.67 (5.11/6.47)	<0.0001
PRP_Peak height (nM), median (25%/75% Q)	97.1 (84.4/119.3)	93.5 (78.3/104.9)	110.8 (89.7/133.6)	<0.0001
PRP_ETP (nM*min), median (25%/75%Q)	1438 (1287/1703)	1403 (1251/1518)	1618 (1362/1823)	<0.0001
PFP_Lag time (min), median (25%/75% Q)	7.50 (6.50/8.83)	8.43 (7.17/9.39)	6.67 (5.72/7.66)	<0.0001
PFP_Peak height (nM), median (25%/75% Q)	79.5 (57.4/123.6)	69.4 (54.3/90.7)	110.8 (73.9/157.6)	<0.0001
PFP_ETP (nM*min), median (25%/75% Q)	765 (589/1067)	662 (536/836)	1005 (734/1338)	<0.0001

Sex-specific clinical and laboratory characteristics of childhood cancer survivors. N, number; %, percentage; SD, standard deviation; 25%/75% Q, 25 percent and 75 percent quartile; LDL, low density lipoprotein (LDL) cholesterol; high density lipoprotein (HDL) cholesterol; HbA1c, hemoglobin A1c; PRP, platelet rich plasma; PFP, platelet free plasma; ETP, endogenous thrombin potential.

The results on vascular function demonstrated that female cancer survivors present with lower reflection index (RI) and stiffness index (SI) compared to male cancer survivors. TG results showed in female cancer survivors shorter lag time, higher peak height and endogenous thrombin potential (ETP) in both platelet rich plasma (PRP) and platelet free plasma (PFP) compared to male cancer survivors, as we previously reported^13^.

Female control subjects presented with lower vascular function measurements compared to male control subjects [RI, mean (standard deviation): females = 59.0% (16.1) vs males = 71.7% (16.2); SI, mean (standard deviation): females = 7.48 m/s (2.48) vs males = 9.65 m/s (3.35)]. The TG results in both PRP and PFP were no different between male and female controls as depicted in Supplemental Table S1 and as previously reported^8^.

The differences for the vascular function and TG parameters between cancer survivors and population-based controls, sex-specific are illustrated in Supplemental Table S2. Both male and female cancer survivors presented with lower RI and SI compared to male and female controls, respectively. Male cancer survivors presented with lower ETP and no different lag time and peak height, assessed in PRP, compared to male controls. The TG profile in PFP showed longer lag time and lower peak height and ETP in male cancer survivors compared to male controls. Differently from males, female cancer survivors presented with shorter lag time and higher peak height and ETP compared to female controls, both in presence and absence of platelets, as we previously reported^13^.

### Correlation between thrombin generation and vascular function

The correlation analysis between TG parameters and RI and/or SI in childhood cancer survivors is presented in Supplemental Fig. S1. An inverse correlation was observed between RI and peak height and/or ETP in both PRP and PFP (in PRP: peak height, Spearman rank correlation coefficient [r_s_] = −0.23; ETP, r_s_ = −0.25; in PFP: peak height, r_s_ = −0.2; ETP, r_s_ = −0.23). The results between TG parameters in PRP and/or PFP and SI, showed no important correlations.

**Figure 1:**
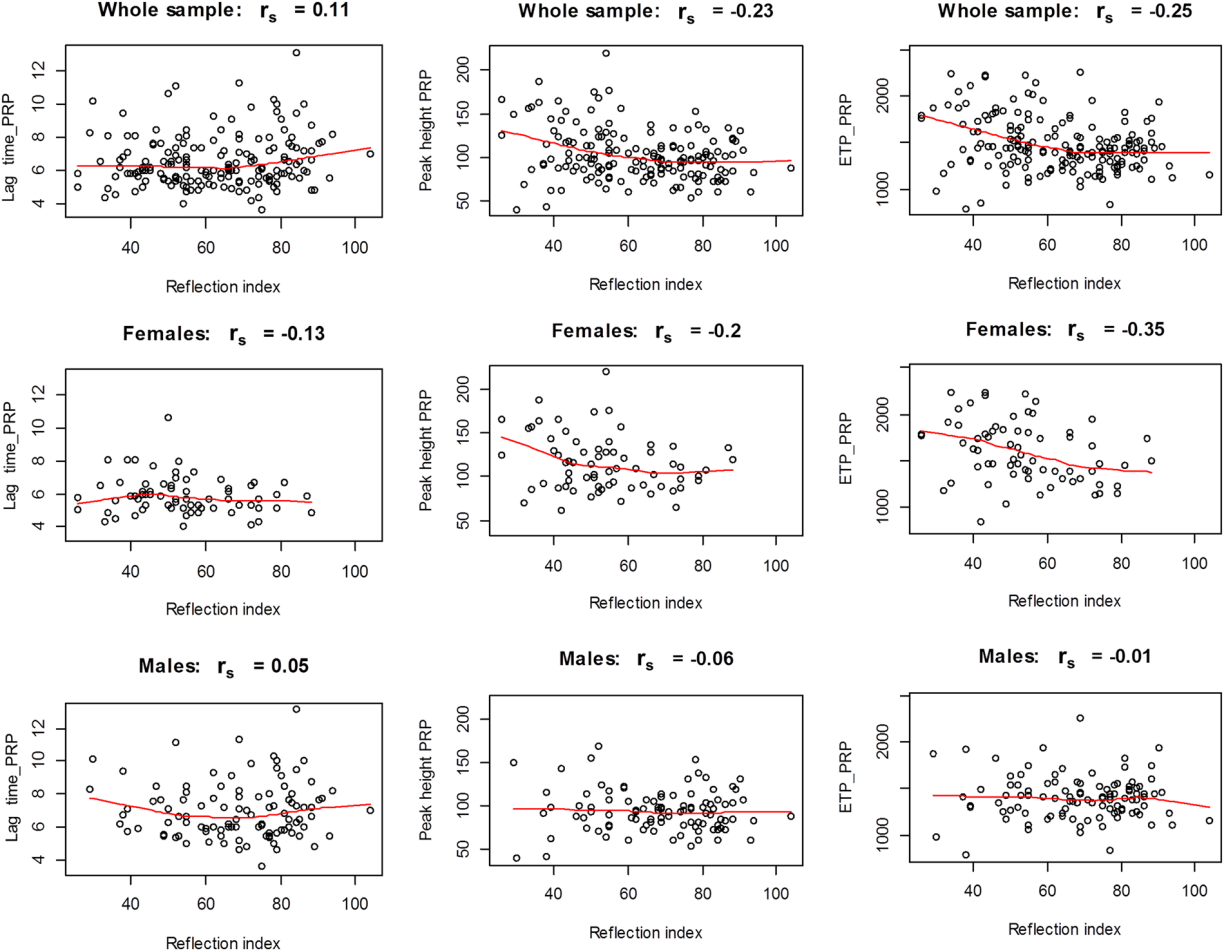
Correlation analysis between platelet-dependent thrombin generation and vascular function in childhood cancer survivors. Presented are scatter plots between lag time and/or peak heigh and/or endogenous thrombin potential (ETP) and reflection index in the whole cancer survivors sample and sex-specific in female and male cancer survivors. PRP, platelet rich plasma; r_s,_ Spearman rank correlation coefficient.

Figure 1 presents a sex-stratified analysis in cancer survivors for the correlations between TG parameters in PRP and RI. In female cancer survivors an inverse correlation between TG parameters and RI (lag time: r_s_ = −0.13, peak height: r_s_ = −0.2 and ETP: r_s_ = −0.35) was observed. In males, no important correlations were observed between TG parameters in presence of platelets and RI.

**Figure 2:**
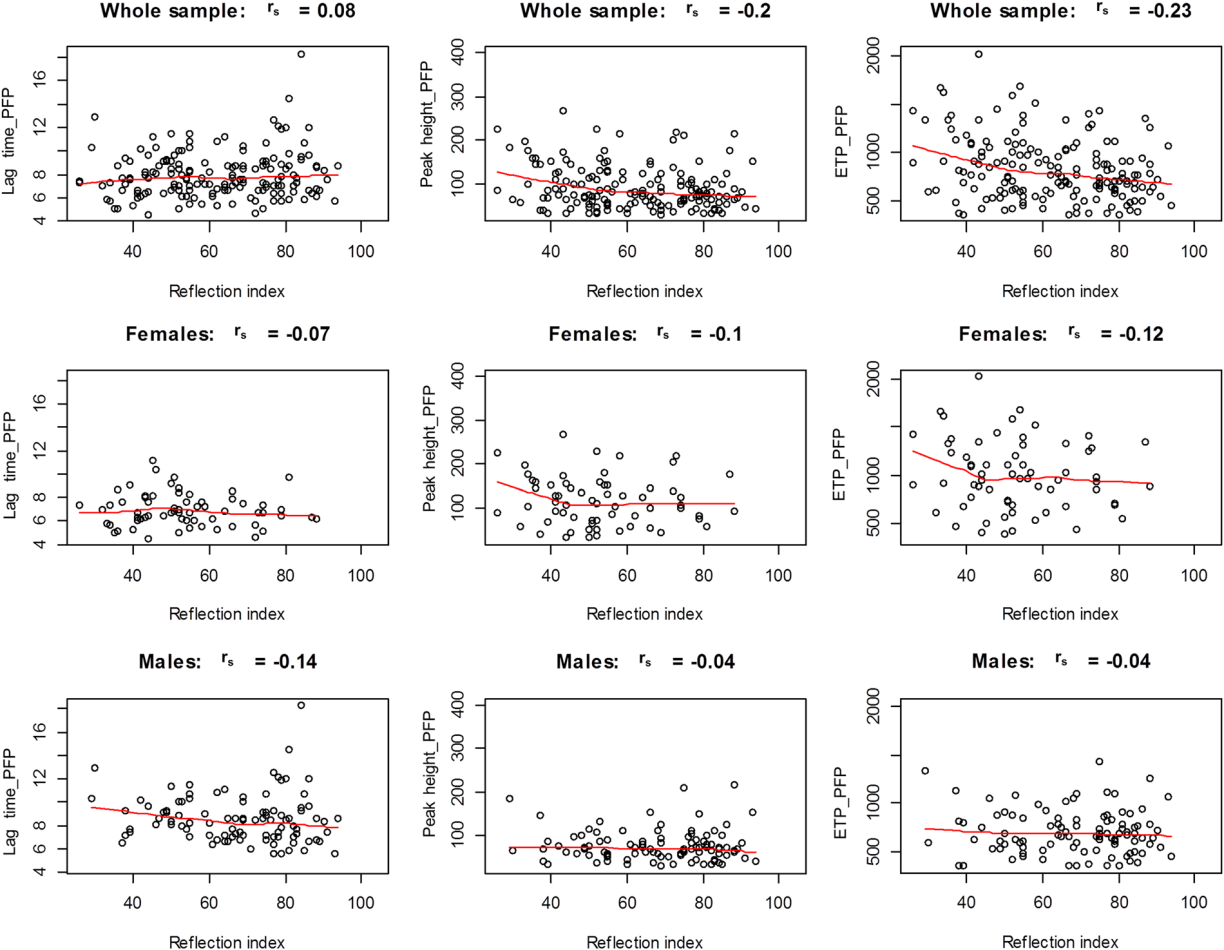
Correlation analysis between thrombin generation in absence of platelets and vascular function in childhood cancer survivors. Presented are scatter plots between lag time and/or peak heigh and/or endogenous thrombin potential (ETP) and reflection index in the whole cancer survivors sample and sex-specific in female and male cancer survivors. PFP, platelet free plasma; r_s_, Spearman rank correlation coefficient.

Figure 2 presents a sex-stratified correlation analysis in cancer survivors between TG parameters in PFP and RI. Whereas in the whole sample a negative correlation was observed between RI and peak height (r_s_ = −0.2) and/or ETP (r_s_ = −0.23), the sex specific analysis showed no relevant correlations between TG parameters in PFP and RI for both female and male cancer survivors.

Supplemental Fig. S2 presents the correlation analysis in control subjects. The results showed no relevant correlations between TG parameters (e.g. lag time, peak height and ETP) and vascular function measurements (e.g. RI and SI).

### Influence of cardiovascular risk factors on the association between platelet coagulant and vascular function in female cancer survivors

The multivariable analysis in female cancer survivors adjusted for age (Table 2) confirmed the inverse association between ETP in PRP and RI (beta estimate, β: −6.85 [95% confidence interval: −11.97, −1.73]). Further adjustment for conventional CVRFs resulted in loss of the observed association (β: −3.30 [−8.17, 1.57]). The effect of traditional CVRFs on the observed association in females between ETP in PRP and RI is shown in Table 3. Adjusting the model for obesity and/or arterial hypertension showed the strongest impact on the estimate of RI for the relation with ETP in PRP. To understand if female hormonal factors further contribute to the observed association between ETP in PRP and RI, an additional adjustment for oral contraceptives and menstrual bleeding was performed (see Table 4). The analysis revealed that these factors did not modify the beta estimate of the RI for the relation with ETP in PRP.

**Table 2:** Association between reflection index and thrombin generation parameters according to presence and absence of platelets in blood plasma of female cancer survivors.

	Lag time (min)		Peak height (nM)		ETP (nM*min)	
RI (%)	PRP	PFP	PRP	PFP	PRP	PFP
Model A Age β (95% CI)	−0.01 (−0.03, 0.01)	−0.01 (−0.03, 0.02)	−0.46 (−0.98, 0.05)	−0.36 (−1.32, 0.59)	−6.85** (−11.97, −1.73)	−3.30 (−9.46, 2.87)
Model B Age + CVRFs β (95% CI)	−0.01 (−0.03, 0.01)	−0.003 (−0.03, 0.02)	−0.32 (−0.89, 0.24)	−0.34 (−1.45, 0.76)	−3.30 (−8.17, 1.57)	−2.89 (−10.02, 4.25)

Multivariable linear regression models in females adjusted for age (Model A) and age plus traditional cardiovascular risk factors (CVRFs) in Model B. Presented are beta estimates (β) and 95 percent (%) of the confidence interval (CI) for change in thrombin generation parameters e.g. lag time, peak height and endogenous thrombin potential (ETP) per unit increase of reflection index (RI) as independent variable. PRP, platelet rich plasma; PFP, platelet free plasma. **p < 0.01.

**Table 3:** Endogenous thrombin potential in platelet rich plasma and reflection index in female cancer survivors – Modulation by cardiovascular risk factors.

Reflection index	ETP in PRP
	β (95% CI)
Age (years)	−6.85** (−11.97, −1.73)
Age + Obesity	−5.55* (−10.29, −0.81)
Age + Smoking	−5.96* (−11.23, −0.69)
Age + HbA1c	−6.54* (−11.56, −1.52)
Age + Arterial Hypertension	−5.28* (−10.42, −0.14)
Age + Dyslipidemia	−6.87** (−11.72, −2.03)
Age + Family history of MI	−6.23* (−11.62, −0.83)
Age + Family history of stroke	−6.48*(−11.80, −1.16)

Multivariable linear regression analysis between endogenous thrombin potential (ETP, dependent variable) measured in platelet rich plasma (PRP) and reflection index (independent variable). The analysis is adjusted for age and subsequently for each cardiovascular risk factor, as presented in the table. Presented data are beta estimate (β) and 95 percent (%) of the confidence interval (CI). *p < 0.05; **p < 0.01; RI, reflection index; HBA1c, haemoglobin A1c; MI, myocardial infarction.

**Table 4:** Effect of female hormonal factors on the relation between endogenous thrombin potential in platelet-rich plasma and reflection index.

Reflection Index	ETP in PRP
Adjustment for confounder	β (95% CI)
Age (years)	−6.85** (−11.97, −1.73)
additionally cardiovascular risk factors	−3.30 (−8.17, 1.57)
additionally oral contraceptives	−3.30 (−8.07, 1.47)
additionally menstrual bleeding	−3.54 (−8.40, 1.31)

Multivariable linear regression analysis in female cancer survivors (n = 85) with endogenous thrombin potential (ETP) in platelet rich plasma (PRP) as dependent variable and reflection index as independent variable. The models are adjusted as presented in the table for age, traditional cardiovascular risk factors (Obesity, Smoking, Diabetes mellitus, Arterial Hypertension, Dyslipidemia and family history of myocardial infarction and/or stroke), oral contraceptives (means regular intake of hormonal drug therapy) and menstrual bleeding (means regular menstrual bleedings). Presented data are beta estimate (β) and 95 percent (%) of the confidence interval (CI). **p < 0.01.

The association between RI with ETP in PRP in female cancer survivors was further evaluated in a multivariable model including cardiovascular relevant laboratory traits as presented in Table 5. Adjustment for the laboratory markers resulted in loss of the observed crude association between RI and ETP in PRP. In addition, the analysis showed that one unit increase (fL) of mean platelet volume (MPV) was associated with 167 nM*min increase in ETP in PRP (p < 0.05). Platelet count showed also a positive association with ETP in PRP in the fully adjusted multivariable model.

**Table 5:** Effect of laboratory traits of cardiovascular risk on association between endogenous thrombin potential in platelet rich plasma and reflection index in female cancer survivors.

	Endogenous Thrombin Potential in PRP β (95% CI)	
Reflection Index (%)	−6.85** (−11.97, −1.73)	−4.06 (−9.16, 1.04)
Erythrocyte (x10^12^/l)		−1.40 (−222.93, 220.12)
Platelets (x10^9^/l)		2.12* (0.33, 3.92)
Leukocyte (x10^9^/l)		−2.83 (−46.87, 41.22)
MPV (fl)		166.97* (13.29, 320.66)
Cholesterol (mg/dl)		−0.86 (−300.55, 298.83)
Tryglicerides (mg/dl)		0.86 (−59.02, 60.73)
HDL-cholesterol (mg/dl)		7.70 (−296.61, 312.02)
LDL-cholesterol (mg/dl)		−1.95 (−300.26, 296.36)
LDL/HDL ratio		191.50 (−183.14, 566.15)
Fasting glucose (mg/dl)		3.72 (−6.74, 14.19)
HbA1c (%)		50.11 (−169.62, 269.85)
C-reactive protein (mg/l)		8.26 (−12.30, 28.81)
Fibrinogen (mg/dl)		0.74 (−0.45, 1.93)

Multivariable linear regression analysis with endogenous thrombin potential in platelet rich plasma (PRP) as dependent variable and reflection index as independent variable adjusted for age in the first column and age plus all variables presented of the second column in the table. Presented data are beta estimate (β) and 95 percent (%) of the confidence interval (CI). MPV, mean platelet volume; HDL, high density lipoprotein; LDL, low density lipoprotein; HbA1c, haemoglobin A1c; **p < 0.01; *p < 0.05.

### Relation between RI and brachial pulse pressure, case in arterial hypertension and obesity

Female cancer survivors presented with lower systolic blood pressure (SBP) and diastolic blood pressure (DBP) compared to male cancer survivors (SBP, mean [standard deviation]: females = 116.8 mmHg [11.9], males = 126.6 mmHg [11.4]; DBP: females = 76.6 mmHg [8.9], males = 79.7 [9.4]). In addition, brachial pulse pressure (bPP) was lower in females compared to male cancer survivors (40.2 mmHg [8.1] vs 46.9 mmHg [7.6]). The correlation analysis between RI and bPP showed no correlation in females (r_s_ = 0.012) and a negative correlation in males (r_s_ = −0.13). Investigating the correlation in subjects with arterial hypertension showed a negative correlation between RI and bPP in both females (r_s_ = −0.35) and males (r_s_ = −0.32). No important correlations between these variables were observed in subjects with obesity (obese females, r_s_ = 0.10; obese males, r_s_ = 0.083).

## Discussion

The general historical notion that men live in better health but shorter and women live in worse health but longer has been challenged in the recent years^14^. Recently, some countries have seen a decrease in the female advantage in life expectancy^15^. Complexity of sex health differences vary according to many aspects of the morbidity process and this is particularly the case in adult survivors of childhood cancer. Despite being recognized that female sex is a risk factor for numerous long-term adverse outcomes in cancer survivors, very little is known about the underlying pathophysiological mechanisms rendering the increased risk^16^. Premature ovarian insufficiency has been reported to contribute to poor general health outcome in female cancer survivors and to increased risk for CVD in the general female population^17,18^.

In the present study, the relation between coagulant and vascular function was investigated sex-specifically in cancer survivors and in population-based control subjects. In our previous work including the same study samples, both cancer survivors and population-based controls, we reported that female survivors presented with higher TG compared to female control subjects. Differently, male cancer survivors showed lower TG profile compared to a male control subjects^13^. The results of the present study showed a strong correlation between thrombin generation and RI, in presence of platelets only. The association between these important functions was found in female cancer survivors only and was modulated by the presence of traditional CVRFs, in particular arterial hypertension and obesity. The female hormonal status reflected by “menstrual bleeding” and “intake of oral contraceptives” showed no effect on the relation between platelet coagulant and vascular function. In female and/or male control subjects no important relation between platelet-coagulant and vascular function was observed. Further, this study finds no important relations for thrombin generation measured in PFP and for arterial SI, for both cancer survivors and population-based control subjects.

SI as a measure of arterial stiffness predominantly reflects vessel structural characteristics, whereas RI as a measure of vascular tone has been mainly dependent on nitric oxide release from the endothelium. Previously published data of the first 5,000 participants of GHS demonstrated a sex difference of the RI with females having lower values compared to males. In addition, that study showed a negative relation between RI and most of the traditional CVRFs such as arterial hypertension and diabetes, including the C-reactive protein as an overall marker of inflammation^19^. The paradoxically lower vascular tone in subjects at risk for cardiovascular disease could be due to preferential stiffening of central (large) arteries leading to decrease in the impedance mismatch in more distal muscular arteries and lower reflection index^20^. A paradoxically lower radial augmentation index has been also reported in subjects with diabetes mellitus and was supported by higher central PP^21^. The present study showed that obesity and arterial hypertension have the largest effect on the relation between platelet dependent TG and RI in female cancer survivors. This study demonstrates a negative correlation between RI and bPP, particularly in subjects with hypertension, supporting the hypothesis of paradoxically lower RI measured on the fourth finger.

We have recently shown that obesity is a major determinant of platelet-coagulant function, both in cancer survivors and population-based control subjects, irrespective of biological sex^8,13^. Hypertension was another important determinant, particularly the high systolic blood pressure in subjects not taking antihypertensive medication, as shown in data from GHS^8^. Differently, in childhood cancer survivors, hypertension-related platelet hypercoagulability was observed in female subjects only^13^. In the present work, higher MPV was suspected as important potential mediator in the relation between platelet TG and RI. Indeed, MPV is becoming an increasingly recognized platelet activation marker, and was linked to both platelet coagulant function and/or augmentation index, another marker of vascular function^8,22^. Interestingly, despite being higher in females, this study showed no effect of fibrinogen and/or CRP on platelet dependent TG.

Platelets can contribute to hypercoagulability and impaired vascular function by several mechanisms. Overweight and particularly obesity have been related with increased platelet reactivity assessed by different laboratory platelet function tests in different series of patients. Increased MPV, platelet microparticles, thromboxane B2 metabolites and thrombin receptor activated peptide-6 induced aggregation are among the platelet function features described in subjects with obesity^23^. Increased oxidative stress with higher reactive oxygen species production, frequently observed in subjects with central obesity, has been attributed as a relevant factor causing platelet dysfunction and nitric oxide (NO) availability^24^. Decreased NO in subjects with obesity could further explain our findings of increased platelet coagulant function. In addition to obesity, arterial hypertension was also an important modulator in the relation between platelet coagulant and vascular function. Hypertension has been well known to induce vascular remodeling with endothelial cell dysfunction including reduced NO availability, oxidative stress, apoptosis and proliferation. In addition, a relation between an angiotensin II-induced hypertension in mice with increased factor XI-dependent platelet coagulant function was reported particularly in conditions of vascular dysfunction^25^. Another recent study further confirms the role of thrombin induced platelet microparticles for endothelial cell proliferation in arterial hypertension^26^.

The present work is the first to show an important relation between platelet coagulant and vascular function, modified by the presence of arterial hypertension and obesity in female cancer survivors. Additionally, as it is still unclear which physiological aspect is captured by different vascular function methods, these data enlighten a potential link between platelet activation and resistant vessel vascular tone. Finally, the results of this study reveal a possible element of the pathophysiological mechanisms that may explain the observed cardiovascular sequelae in female cancer survivors.

## Methods

### Study design and participants

The CVSS study is an epidemiological, observational study designed to investigate German childhood cancer survivors who had a neoplasia according to the International Classification of Childhood Cancer (ICCC 3)^27^ prior to the age of 15 years between 1980 and 1990 with respect to cardiovascular health, as described in detail before^12^. Survivors with second malignant neoplasia were excluded from analysis. Adult cancer survivors, treated in Mainz and treatment centers within a radius of approximately 300 kilometres, were invited. All participants pursued a 5-hour examination at the study center following standard operating procedures by certified medical technical assistants. The subjects for the present substudy (n = 200) were randomly selected from the CVSS cohort in the period between February 2015 and January 2016, as reported recently^13^. Assessment and definition of conventional CVRFs, CVD and categorization of current medication intake are described in part A of the Supplemental Material. From the population-based GHS, a subsample of n = 407 individuals were randomly selected between February 2015 and October 2016. Individuals with cancer history and intake of anticoagulants were excluded, as described elsewhere in more detail^8,13^, resulting in n = 335 subjects as a control group. The study protocol was approved by the Ethics Committee of the State Chamber of Physicians of Rhineland-Palatinate, Germany (for GHS Ref.No. 837.020.07(5555)) and the study project was approved by the steering committee of the GHS and CVSS, as previously declared^12^. The study has been conducted in accordance with the Declaration of Helsinki. Written informed consent was obtained from all participants before entering the study^12,13^.

### Blood sampling and plasma preparation

Venous blood sampling was performed using tubes containing K3-ethylenediaminetetraacetic acid (EDTA) for routine biochemistry analysis including blood cell count, or trisodium citrate (0,129 M, 1:9 vol:vol) for investigation of thrombin generation. Samples were collected under standardized conditions according to standardized operating procedures, as previously reported^13^. All subjects were in fasting state for at least 11 hours before the blood draw^12^. Plasma preparation was performed according to the recommendations for TG measurements, as described elsewhere^13,28^. PRP was isolated by centrifugation of whole blood for 10 min at 200 x g at room temperature. The rest of the blood sample was centrifuged at 2,000 x g for 15 min to obtain platelet poor plasma that was utilized to dilute platelet-rich plasma in order to obtain 150,000 platelets/µl. PFP was isolated by double centrifugation: First, the blood sample was centrifuged for 5 min at 2,000 x g at room temperature. Second, the obtained plasma was centrifuged again for 10 min at 10,000 x g. The isolated PFP was stored at −80 °C until lab testing.

### Standard laboratory measurement

Blood cell counts were automatically determined on an ADVIA 120 Hematology System (Siemens, Erlangen, Germany) between 30 to 90 minutes after blood sampling. Biochemical analysis of blood glucose, CRP and fibrinogen measured in plasma and cholesterol, HDL, LDL and triglycerides measured in serum were determined on the day of sampling by routine methods. All standard laboratory measurements were performed in the Institute of Clinical Chemistry and Laboratory Medicine of the University Medical Center in Mainz, as previously reported^8,13^.

### Thrombin generation measurement

Measurement of TG was performed using the calibrated automated thrombogram (CAT) assay (Thrombinoscope BV, Maastricht, The Netherlands) according to previously described standardized protocols in the platelet epidemiology laboratory of the Center for Thrombosis and Hemostasis (CTH), University Medical Center Mainz, Germany^29,30^. TG in PRP was assessed in fresh material whereas in PFP in stored material of the same individuals. For TG measurements in PRP, adjusted with autologous platelet poor plasma to 150,000 platelets/μl, coagulation was triggered by adding 20 μl exogenous low tissue factor (1pM) to 80 μl PRP. For measurements in PFP, 20 μl exogenous low tissue factor together with 4 μM phospholipids were added to 80 μl plasma. After 10 minutes prewarming at 37 °C in the fluorimeter, the reaction was started by adding 20 μl of a low-affinity fluorogenic substrate for thrombin (Z-Gly-Gly-Arg-AMC) and calcium chloride mixture (FluCa). To correct for inner filter effects and substrate consumption, TG measurements were calibrated against a signal from the calibration well obtained in a sample from the same plasma (80 μL platelet-rich or platelet-free plasma, respectively) supplemented with a fixed amount of thrombin–α2-macroglobulin complex (20 μL of Thrombin Calibrator) and 20 μL of FluCa by means of Thrombinoscope software (Thrombinoscope BV), as described before^13^. All CAT reagents were obtained from Stago Deutschland GmbH (Düsseldorf, Germany). Lag time as time to minimum thrombin formed expressed in minutes (min), peak height as maximum concentration of thrombin formed expressed in nM thrombin and ETP or area under the curve expressed as nM per minute thrombin formed were the investigated TG parameters of interest^28^.

### Vascular function measurements

The digital volume pulse was continuously obtained by measuring the transmission of infrared light through the finger pulp of the right ring (or fourth) finger with a PulseTrace 2000 device (Micro Medical Limited; Rochester, UK). The reflection index, a measure of vascular tone, was automatically calculated as the relative amplitude of the forward wave (early systolic peak) and reflected wave component (diastolic peak). The time difference between the two waves is called peak-to peak time (PPT) and was used to calculate the arterial stiffness index with the following formula: subject height in meter/PPT in seconds (m/s). The measurements were performed according to previously described standardized protocols in the GHS center, University Medical Center Mainz, Mainz, Germany^31^.

### Statistical analysis

All clinical and laboratory data of the present investigation underwent quality control by a central data management unit. Data were reviewed for completeness by predefined algorithms and plausibility criteria, as reported in our previous work with the same study population^13^. Normally distributed continuous variables were described using mean ± standard deviation (SD) and tested with T-test, skewed variables were described using median and 25 percent and 75 percent quartile and tested with the Mann-Whitney *U*-test. Categorical variables are expressed as absolute numbers and percentages and tested with Chi-squared test. Relations between continuous variables were explored by Spearman rank correlation coefficient (r_s_). Multivariable linear regression analysis was used to assess the associations between TG parameters, i.e. ETP, as dependent variable and vascular function measurements, i.e RI and SI, as independent variables in models adjusted for age and conventional CVRFs. Because of only three subjects with diabetes, haemoglobin A1c (HbA1c) was used instead in the regression models. P values should be interpreted as a continuous measure of statistical evidence and no significance threshold was defined because of the explorative character of the analysis. Statistical analysis was performed with software program R, version 3.4.3 (http://www.R-project.org).

## Supplementary information

Supplemental Material
