# Supplementary material for: Relation between platelet coagulant and vascular function, sex-specific analysis in adult survivors of childhood cancer compared to a population-based sample
Source: Sci Rep. 2019 Dec 27;9:20090. doi: 10.1038/s41598-019-56626-1 (PMC6934665; doi:10.1038/s41598-019-56626-1)
Supplement: Supplementary file 1 — Supplemental Material [file 41598_2019_56626_MOESM1_ESM.doc]

**Supplemental Material**

**Relation between platelet coagulant and vascular function, sex-specific analysis in adult survivors of childhood cancer compared to a population-based sample**

Marina Panova-Noeva^1,2,7^, Bianca Wagner^2^, Markus Nagler^2^, Natalie Arnold^2,7^, Jürgen H. Prochaska^1,2,7^, Susan Eckerle^3^, Henri M. Spronk^4^, Hiltrud Merzenich^5^, Arthur Wingerter^3^, Astrid Schneider^5^, Sven Danckwardt^1,6,7^, Hugo ten Cate^4,1^, Jörg Faber^3^, Philipp S. Wild^2,1,7^

^1^ Center for Thrombosis and Hemostasis (CTH), University Medical Center of the Johannes Gutenberg-University Mainz, Germany

^2^ Preventive Cardiology and Preventive Medicine, Center for Cardiology, University Medical Center of the Johannes Gutenberg-University Mainz, Germany

^3^ Department of Pediatric Hematology/Oncology/Hemostaseology, Center for Pediatric and Adolescent Medicine, University Medical Center of the Johannes Gutenberg-University Mainz, Germany

^4^ Laboratory for Clinical Thrombosis and Hemostasis, Department of Internal Medicine, Cardiovascular Research Institute Maastricht (CARIM), Maastricht University Medical Center, the Netherlands

^5^ Institute of Medical Biostatistics, Epidemiology and Informatics (IMBEI) at the University Medical Center Mainz, Germany

^6^ Institute for Clinical Chemistry and Laboratory Medicine, University Medical Center of the Johannes Gutenberg-University Mainz, Germany

^7^ DZHK (German Center for Cardiovascular Research), Partner Site RhineMain, Mainz, Germany

**Part A. Supplemental Methods**

**Definition of classical cardiovascular risk factors and cardiovascular disease**

Obesity has been defined as a body-mass index ≥ 30 kg/m². Individuals have been classified as smokers (daily smokers ≥1 cigarette/day and occasional smokers <1 cigarette/day), former smokers and non-smokers (never smoked). Diabetes mellitus, dyslipidemia and hypertension have been defined in individuals with definite diagnose by a physician. In addition, diabetes mellitus has been defined if a blood glucose level of ≥126mg/dL in the baseline examination after an overnight fast of at least 8 hours or a blood glucose level of ≥200mg/dL in the baseline examination after a fasting period >5 hours. Dyslipidemia has been additionally defined if low-density lipoprotein/high-density lipoprotein ratio of >3.5 and/or triglycerides level ≥150mg/dl. Individuals taking antihypertensive drugs or having a mean systolic blood pressure of ≥140mmHg or a mean diastolic blood pressure of ≥90mmHg (in the 2nd and 3rd standardized measurement after 8 and 11 minutes of rest) have been also defined with hypertension. Self-reported coronary artery disease (CAD), myocardial infarction (MI), heart failure (HF), stroke, deep vein thrombosis (DVT), pulmonary embolism (PE) and peripheral arterial disease (PAD) indicated personal history of cardiovascular disease. Positive family history was defined as myocardial infarction and/or stroke of male first-degree relatives until the age of 60 years or female first-degree relatives until the age of 65 years.

**Categorization of medication**

Medications that the study participants were taking were registered on site at the GHS study center by scanning the bar codes from the original packages of the drugs or by personal documentation (drug lists) if not available. The medication was classified according to the Anatomical Therapeutic Chemical (ATC) classification system. The following medication groups were selected for analysis: antithrombotic agents (B01A), aspirin (B01AC56), antilipemic drugs (C10A), antihypertensive drugs (C02), angiotensin converting enzyme (ACE) inhibitor (C09), diuretics (C03), angiotensin receptor blocker (ARBs) (C09C), calcium channel blocker (C08) and B-blocker (C07). For the use of oral contraceptives and/or hormone replacement therapy self-reported information was used.

**Part B. Supplemental Table**

**Table S1. Characteristics from a population-based control sample**

|  | **Whole sample** | **Males** | **Females** | **p-value** |
| --- | --- | --- | --- | --- |
| Number (N) | 335 | 170 | 165 | - |
| Sex (females), % (N) | 49.2% (165) | - | - | - |
| Age (years), mean (SD) | 57.9 (10.4) | 57.6 (10.1) | 58.2 (10.6) | 0.62 |
| **Cardiovascular Risk Factors** | | | | |
| Obesity, % (N) | 25.4% (85) | 27.6% (47) | 23.0% (38) | 0.38 |
| Smoking, % (N) | 16.4% (55) | 14.1% (24) | 18.8% (31) | 0.30 |
| Diabetes, % (N) | 10.4% (35) | 13.5% (23) | 7.3% (12) | 0.074 |
| Hypertension, % (N) | 50.4% (169) | 52.4% (89) | 48.5% (80) | 0.51 |
| Dyslipidemia, % (N) | 43.9% (147) | 51.8% (88) | 35.8% (59) | 0.0041 |
| **Standard laboratory** | | | | |
| Platelet count (x10^9^/l), mean (SD) | 235 (56) | 221 (53) | 249 (56) | <0.0001 |
| Mean Platelet Volume (fl), median (25%/75% Q) | 7.60 (7.20/8.18) | 7.60 (7.10/8.20) | 7.60 (7.20/8.13) | 0.77 |
| Leukocyte count (x10^9^/l), mean (SD) | 6.70 (1.74) | 6.60 (1.71) | 6.80 (1.76) | 0.28 |
| Cholesterol (mg/dl), mean (SD) | 226 (42) | 217 (40) | 235 (43) | <0.0001 |
| Triglycerides (mg/dl), median (25%/75% Q) | 110.0 (83.2/149.7) | 116.0 (87.9/156.1) | 102.0 (81.0/133.3) | 0.024 |
| LDL/HDL ratio, mean (SD) | 2.62 (0.94) | 2.85 (0.95) | 2.38 (0.87) | <0.0001 |
| HbA1c (%), median (25%/75% Q) | 5.60 (5.40/5.80) | 5.60 (5.30/5.90) | 5.60 (5.40/5.80) | 0.67 |
| C-reactive protein (mg/l), median (25%/75% Q) | 1.40 (0.77/3.27) | 1.40 (0.77/2.80) | 1.40 (0.80/3.70) | 0.39 |
| Fibrinogen (mg/dl), mean (SD) | 319 (69) | 305 (68) | 333 (67) | 0.00027 |
| **Vascular Function** | | | | |
| Reflection index (%), median (25%/75% Q) | 68.0 (52.0/80.0) | 77.0 (60.2/83.0) | 59.0 (48.0/71.7) | <0.0001 |
| Stiffness index (m/s), median (25%/75% Q) | 7.63 (6.06/10.75) | 9.36 (6.58/12.18) | 6.56 (5.66/8.55) | <0.0001 |
| **Thrombin Generation** | | | | |
| PRP_Lag time (min), median (25%/75% Q) | 6.67 (5.72/7.75) | 6.67 (5.83/7.58) | 6.67 (5.63/7.85) | 0.59 |
| PRP_Peak height (nM), median (25%/75% Q) | 94.7 (77.2/111.1) | 96.8 (79.1/111.6) | 92.8 (75.8/110.3) | 0.27 |
| PRP_ETP (nM*min), median (25%/75% Q) | 1468 (1339/1640) | 1469 (1353/1638) | 1450 (1320/1649) | 0.81 |
| PFP_Lag time (min), median (25%/75% Q) | 4.50 (4.00/5.00) | 4.50 (4.00/5.00) | 4.50 (4.00/5.17) | 0.86 |
| PFP_Peak height (nM), median (25%/75% Q) | 92.0 (70.8/111.8) | 93.6 (75.8/111.9) | 87.6 (68.2/111.6) | 0.24 |
| PFP_ETP (nM*min), median (25%/75% Q) | 814 (650/964) | 827 (676/956) | 794 (640/966) | 0.18 |

Sex-specific clinical and laboratory characteristics of Gutenberg Health Study participants. N, number; %, percentage; SD, standard deviation; 25%/75% Q, 25 percent and 75 percent quartile; LDL, low density lipoprotein (LDL) cholesterol; high density lipoprotein (HDL) cholesterol; HbA1c, hemoglobin A1c; PRP, platelet rich plasma; PFP, platelet free plasma; ETP, endogenous thrombin potential.

**Table S2. Vascular function and thrombin generation parameters between cancer survivors and population-based controls**

|  | **Males** | | | **Females** | | |
| --- | --- | --- | --- | --- | --- | --- |
|  | **GHS** | **CVSS** | **p-value** | **GHS** | **CVSS** | **p-value** |
| Number | 170 | 115 | - | 165 | 85 | - |
| Age (years), mean (SD) | 57.6 (10.1) | 35.5 (5.4) | <0.0001 | 58.2 (10.6) | 34.6 (5.1) | <0.0001 |
| Reflection index (%), median (25%/75% Q) | 77.0 (60.2/83.0) | 71.0 (55.0/81.0) | 0.054 | 59.0 (48.0/71.7) | 52.0 (43.0/64.2) | 0.011 |
| Stiffness index (m/s), median (25%/75% Q) | 9.36 (6.58/12.18) | 6.65 (5.95/8.26) | <0.0001 | 6.56 (5.66/8.55) | 5.76 (5.01/7.02) | <0.0001 |
| PRP_Lag time (min), median (25%/75% Q) | 6.67 (5.83/7.58) | 6.78 (5.83/8.00) | 0.22 | 6.67 (5.63/7.85) | 5.67 (5.11/6.47) | <0.0001 |
| PRP_Peak height (nM), median (25%/75% Q) | 96.8 (79.1/111.6) | 93.5 (78.3/104.9) | 0.15 | 92.8 (75.8/110.3) | 110.8 (89.7/133.6) | <0.0001 |
| PRP_ETP (nM*min), median (25%/75% Q) | 1469 (1353/1638) | 1403 (1251/1518) | 0.00062 | 1450 (1320/1649) | 1618 (1362/1823) | 0.013 |
| PFP_Lag time (min), median (25%/75% Q) | 4.50 (4.00/5.00) | 8.43 (7.17/9.39) | <0.0001 | 4.50 (4.00/5.17) | 6.67 (5.72/7.66) | <0.0001 |
| PFP_Peak height (nM), median (25%/75% Q) | 93.6 (75.8/111.9) | 69.4 (54.3/90.7) | <0.0001 | 87.6 (68.2/111.6) | 110.8 (73.9/157.6) | 0.0012 |
| PFP_ETP (nM*min), median (25%/75% Q) | 827 (676/956) | 662 (536/836) | <0.0001 | 794 (640/966) | 1005 (734/1338) | <0.0001 |

The table is presenting the vascular function and thrombin generation results compared between CVSS and GHS participants, sex-specifically. GHS, Gutenberg Health Study; CVSS, Cardiac and Vascular late Sequelae in long-term Survivors of childhood cancer study; PRP, platelet rich plasma; ETP, endogenous thrombin potential; PFP, platelet free plasma. SD, standard deviation, 25%/75% Q, 25 percent and 75 percent quartile.

**Part C. Supplemental Figure**

**Figure S1. Correlation analysis between thrombin generation and vascular function parameters in cancer survivors.**

**
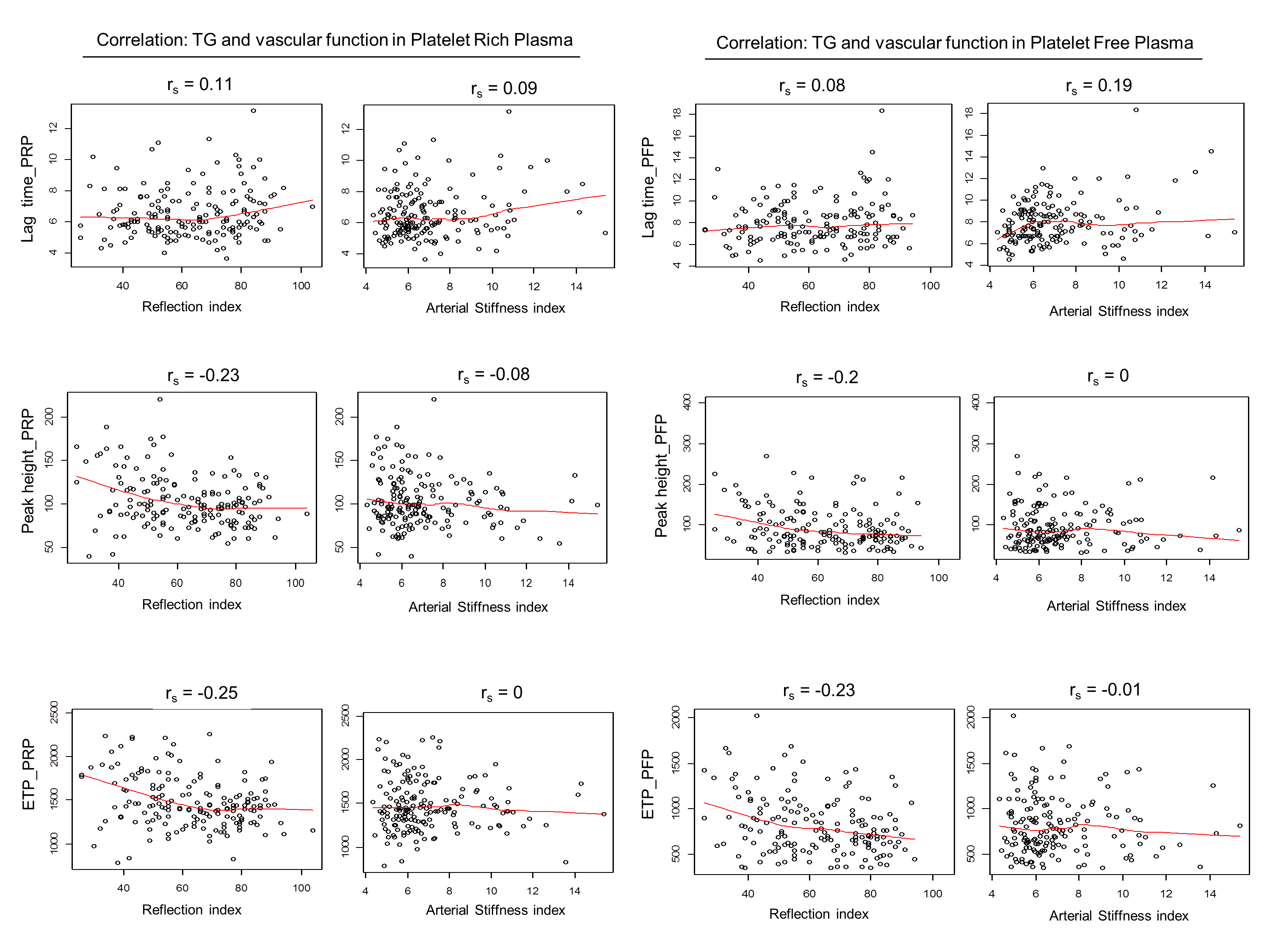
**

Abbreviations: TG, thrombin generation; PRP, platelet rich plasma; PFP, platelet free plasma; ETP, endogenous thrombin potential; r_s_, Spearman rank correlation coefficient.

**Figure S2.** Correlation analysis between thrombin generation and vascular function parameters in a population-based control sample.


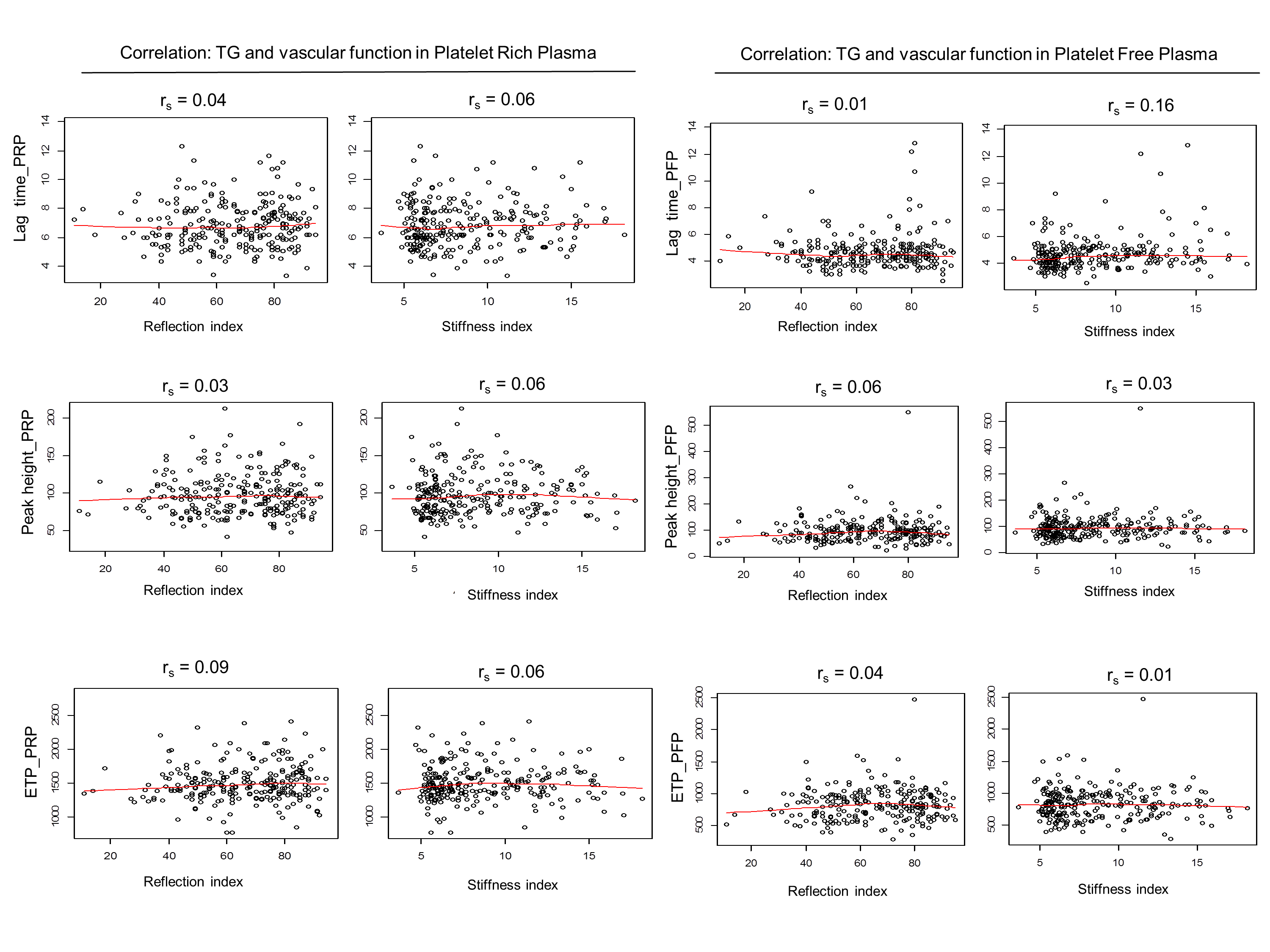


Abbreviations: TG, thrombin generation; PRP, platelet rich plasma; PFP, platelet free plasma; ETP, endogenous thrombin potential; r_s_, Spearman rank correlation coefficient.
